# Comparison of hyperdry amniotic membrane transplantation and conjunctival autografting for primary pterygium

**DOI:** 10.1186/s12886-018-0784-4

**Published:** 2018-05-15

**Authors:** Xin Pan, Daguang Zhang, Zhifang Jia, Zhehui Chen, Yuetian Su

**Affiliations:** 1 The Second Hospital of Jilin University, No.218, Ziqiang Road, Changchun, 130041 China; 2 The First Hospital of Jilin University, No.71, Xinmin Road, Changchun, 130021 China

**Keywords:** Primary pterygium, Hyperdry amniotic membrane transplantation, Conjunctival autografting, Recurrence rate

## Abstract

**Background:**

The purpose of this study was to evaluate the safety and effectiveness of the hyperdry amniotic membrane transplantation compared with conjunctival autografting for the treatment of primary pterygium.

**Methods:**

One hundred and forty-one eyes from 130 patients with primary pterygium were treated with excision followed by hyperdry amniotic membrane or conjunctival autografting after random selection. Seventy-nine eyes from 71 patients received hyperdry amniotic membrane transplantation (HD-AM group), and 62 eyes from 59 patients received conjunctival autografting (CG group). Patients were followed up at one week and one, three, six, and 12 months post-surgery. Recurrence rate, postoperative complications, and final follow-up patient visits were prospectively evaluated.

**Results:**

The mean follow-up duration was 12.56 ± 4.35 months in the HD-AM group and 12.85 ± 3.90 months in the CG group. Recurrences were detected in four eyes (5.06%) in the HD-AM group and 13 eyes (20.97%) in the CG group. A statistically significant difference in frequency of recurrence between the two groups (*P* = 0.003) was observed. The cumulative non-recurrence rates at six and 12 months in all patients stratified by age and sex were not significantly different (*P* = 0.642 and *P* = 0.451, respectively, by log-rank test). Graft retraction and necrosis were not detected in the two groups during the follow-up period.

**Conclusion:**

Hyperdry amniotic membrane transplantation was effective in preventing pterygium recurrence when compared with conjunctival autografting and can be considered a preferable and safe grafting procedure for primary pterygium.

**Trial registration:**

Current Controlled Trials ISRCTN16900270, Retrospectively registered (Date of registration: 3 May 2018).

## Background

Pterygium, a common benign ocular surface lesion, is a wing-shaped fibrovascular growth arising from subconjunctival tissue extending across the nasal limbus onto the cornea that can cause vision loss [1]. Epidemiologic studies indicate that the high rate of pterygium is strongly related to chronic exposure to ultraviolet radiation, dryness, heat, wind, dust, viruses, and oncogenes [2]. Surgical excision is considered the conventional definitive treatment of pterygium [3]. The mainstay techniques presently in use include bare sclera excision followed by adjunctive mitomycin C and β-irradiation or covering the defect with graft tissue such as a conjunctival autograft and amniotic membrane (AM). The major problem associated with pterygium surgery is the incidence of recurrence, but none of the techniques have achieved complete success in completely preventing this recurrence. Currently, conjunctival autografting is the most commonly used technique with a lower recurrence rate and fewer complications despite the requirement for more technically demanding surgical skills and experience; it is more time-consuming to perform [4–6]. Furthermore, it is not feasible to cover large defects created in large pterygia.

Hyperdry (HD)-AM was developed as a new matrix material that is suitable for tissue engineering applications in the form of a surgical patch [7]. It is a new type of AM that is expanded on a nitrocellulose filter paper with epithelial sheet facing upward, processed using consecutive far-infrared rays and microwaves (patented hyperdry method), and sterilized by cobalt-60 irradiation [8]. Thereafter, it was cut into all kinds of squares, vacuum packed, and stored safely at room temperature [9]. Recently, HD-AM has been exploited as a new ophthalmic tool for the management of many ocular surface diseases, including corneal perforations and bleb leaks [10].

This study was intended to evaluate the recurrence of primary pterygium after HD-AM transplantation compared with conjunctival autografting. To the best of our knowledge, this is the first report on the use of HD- AM for treatment of pterygium.

## Methods

### Study group

One hundred and forty-one eyes from 130 patients with primary pterygium were enrolled in this study between March 2015 and February 2016. In all cases, the size of the pterygium was at least 2 mm onto the cornea or causing extreme irritation. Most of the pterygium was translucent, and the episcleral vessels underneath the body of the pterygium could be identified, as previously reported by Tan [11]. Exclusion criteria included recurrent pterygium, dry eye, infection and inflammation of ocular area, glaucoma, and previous ocular surgery in the study eye. Patients were randomized into the hyperdry amniotic membrane transplantation or conjunctival autografting groups (HD-AM and CG groups, respectively) for pterygium excision. Informed consent for the surgery was signed by all patients. Patients with a < 6-month follow-up period were excluded.

### Surgical methods

Pterygium excision: All surgical procedures were performed by the same surgeon using an operating microscope (Zeiss, Germany). After injection of 2% lidocaine hydrochloride containing 1:10000 adrenaline (epinephrine) into the body of the pterygium, the conjunctival sac was irrigated with gentamicin, and the lid speculum was inserted. The head was separated and removed from the cornea by blunt dissection. Residual tissue over the corneal defect area was shaved with toothed forceps. Subconjunctival fibrous tissue under the pterygium was removed as much as possible avoiding damage to the underlying muscle sheath. A rectangular area of bare sclera was created to which the graft could be directly attached.

**Fig. 1:**
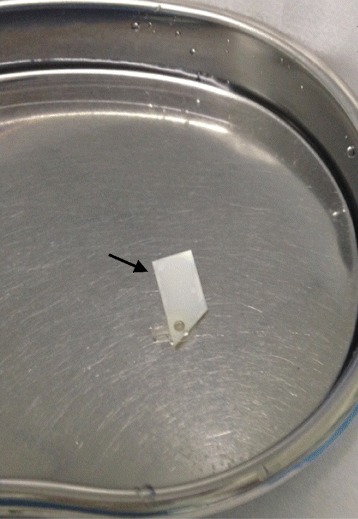
The hyperdry amniotic membrane (HD-AM) (arrow) was expanded on a nitrocellulose filter paper and was rinsed in physiological saline

Hyperdry amniotic membrane transplantation: The hospital ethics committee approved the use of HD-AM in pterygium surgery (2015 No. 063). After the preserved biological amniotic membrane (Jiangxi Ruiji BOI-Engineering Technology Co. Ltd.) was rinsed in physiological saline for 15 min (Fig. 1), it was cut into an appropriate size with scissors, peeled from the filter paper, and placed over the bare sclera area with epithelial basement membrane side facing up. The free edge of the HD-AM was sutured through the episcleral tissue to the edge of conjunctiva along the bare sclera border with 10–0 nylon sutures interrupted and was tightly pressed centrally to securely attach it to the bare sclera. The membrane was placed over the corneal lesion.

Conjunctival autograft transplantation: A conjunctival free graft of similar size was obtained from the superotemporal bulbar conjunctiva by splitting at the anatomic limbus. Careful excision was given to obtain a thin, tenon-free conjunctival graft. The limbal side of the autograft was sutured to the limbal side of the bare scleral bed by separate 10/0 nylon sutures. The donor site was later closed with a continuous suture of 10–0 nylon sutures.

Post-operation and Follow-up: Postoperatively, all the patients received 0.1% fluorometholone (Santen, Osaka, Japan) and tobramycin (Alcon) drops four times daily. The drops were gradually tapered back within one month. Sutures were removed after one week. The patients were examined on the first postoperative day, followed by the first week and then one, three, six, and 12 months postoperatively. The minimum follow-up time was six months. The recurrence rate of pterygium after surgery was used as the primary outcome in this study. Recurrence was defined as the regrowth of the fibrovascular proliferation tissue invading the cornea again. Other complications such as pyogenic granuloma, inclusion cyst, or scleral thinning were recorded.

### Statistical analyses

Statistical analysis was conducted using SPSS 18.0 software. The data were presented as means (±SD) or frequencies (%). χ^2^ test was applied to compare the categorical data between the two groups and frequency of recurrence, whereas the unpaired *t*-test was used to analyze continuous variables such as age and graft size and follow-up times. The cumulative proportion of recurrence was analyzed by the Kaplan–Meier method and log-rank test. Significance was set at *P* < 0.05 using two-sided comparisons.

## Results

The characteristics of patients in the two groups were compared in Table 1. There were no statistically significant differences regarding sex (*P* = 0.963), age (*P* = 0.549), and laterality (*P* = 0.973) between the two groups. The mean follow-up period was 12.56 ± 4.35 months for the HD-AM group and 12.85 ± 3.90 months for the CG group (*P* = 0.674). The size of the extension placed onto the cornea was 3.685 ± 0.848 mm in the HD-AM group and 3.469 ± 0.970 mm in the CG group (*P* = 0.164).

**Table 1:** Comparison of patients’ demographic data among amniotic membrane graft group and conjunctival autograft group

	HD-AM	CG	*P*
No. of patients (eyes)	71(79)	59(62)	–
Sex (M:F)	31:40	26:33	0.963
Mean age (SD)	62.32(7.030)	63.05(6.678)	0.549
Laterality (R:L)	38:41	30:32	0.973
Corneal extension (mm) (Range)	3.685 (0.848) (1.5–5.5)	3.469 (0.970) (1.0–5.6)	0.164
Mean follow up (SD)	12.56(4.35)	12.85 (3.90)	0.674
(Range)	(6–28)	(6–27)	
No. of recurrences (%)	4/79(5.06)	13/62 (20.97)	0.003

*M* male, *F* female, *R* right, *L* left, *FU* follow-up

**Fig. 2:**
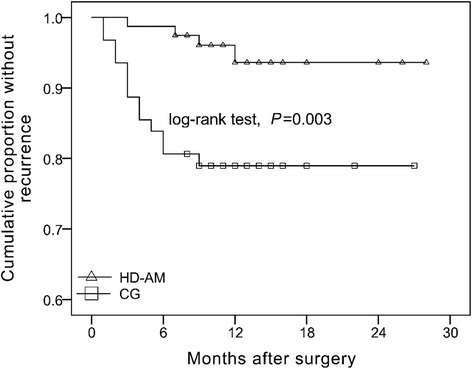
Kaplan-Meier survival curve of recurrence after pterygium excision. The cumulative proportion recurrence-free at 12 months was 0.95 ± 0.03 in the HD-AMT group and 0.78 ± 0.05 in the CG group (P = 0.003)

Four of 79 eyes (5.06%) in the HD-AM group developed pterygium recurrence compared with 13 of 62 eyes (20.97%) in the CG group (*P* = 0.003). In the HD-AM group, four eyes had recurrence at three, seven, nine, and 12 months (mean, 7.75 months) postoperatively. In the CG group, all recurrence developed within six months (mean, 3.4 months) postoperatively. The cumulative recurrence-free proportions at 12 months were 0.95 ± 0.03 in the HD-AM group and 0.78 ± 0.05 in the CG group, which were significantly different (P = 0.003) (Table 2 and Fig. 2). When stratified regardless of surgical groups, there were no significant differences in recurrence rates among patients < 55 years (four cases, 19.05%), between 55 and 65 years (seven cases, 10.95%), and > 65 years (six cases, 10.71%, *P* = 0.642). Also, when stratified by sex only, there were no significant differences in the recurrence rates between male (eight cases, 10.62%) and female patients (nine cases, 14.29%, *P* = 0.45; Table 3).

**Table 2:** Comparison of cumulative non-recurrence rate among hyperdry amniotic membrane transplantation graft group and conjunctival autograft group

		Cumulative non-recurrence rate (%)		*P* Value
		6 months	12 months	
Hyperdry amniotic membrane transplantation graft		98.73	95.42	0.003
conjunctival autograft		83.83	77.81

**Table 3:** Comparison of cumulative non-recurrence rate for all patients stratified by age and sex only

	No. (%) of recurrences	Cumulative non-recurrence rate (%)		
		6 months	12 months	*P* Value
Age (years)				
< 55	4/21(19.05)	95.24	84.35	0.642
55–65	7/64 (10.94)	92.19	88.00	
> 65	6/56(10.71)	91.07	88.77	
Sex				
Male (eye)	8/78 (10.62)	90.48	84.01	0.415
Female (eye)	9/63 (14.29)	93.53	90.42	

Three cases of Dellen ulcer and two cases of Tenon’s cyst were observed in the CG group in the first postoperative week. Dellen cases were treated medically, and Tenon’s cyst was punctured to drain the subconjunctival fluid or excised surgically. No complications were observed in the HD-AM group.

## Discussion

Today, surgical excision is still main method for treating pterygium [5, 12]. The major and most important criteria for the success of the surgery is the rate of postoperative pterygium recurrence, which has been described as the development of fibro-vascular tissue on to the excision site [13]. In order to lower the recurrence rate, the use of antimetabolites has been suggested owing to their antifibrotic and antiangiogenic properties [14, 15].

Conjunctival autografting was first described in 1980 with recurrence rates reported to be between 2.9–39% in primary pterygium [16–20]. In this study, the recurrence rate was 20.97% after conjunctival autografting in the primary pterygium when compared with work done by other researchers. Variations in the recurrence rates of conjunctival autografting could be explained according to the surgical excision size, surgeon’s experience, patient’s age, and surgical technique. As for surgical technique, incomplete separation of Tenon’s tissue from the graft can cause graft retraction and high recurrence rates [17].

While conjunctival autografting has gained worldwide acceptance for treatment of pterygium, it is not without its defects such as the long operation time, graft inversion, and iatrogenic injury to the rest of the conjunctiva [19]. Syam et al. found that 36.66% of patients developed conjunctival scarring at the site of the donor conjunctiva [21]. Therefore, it is not feasible to use this technique to cover wide ocular surface defects created in the cases of large or double-headed pterygia.

HD-AM is made with fresh human AM using the hyperdrying method and returns to a layered structure similar to that of fresh AM after absorbing water as shown in Fig. 1 [7, 8]. Okabe et al. found that the structures of collagen fibers in the connective tissues were not destroyed by the hyperdry device and were more stable than cryopreserved AM [7]. Allen et al. also showed that the biochemical composition of the dried AM, including the number of factors such as epidermal growth factor and TGF-β1, were similar to fresh AM [22]. Moreover, rabbit models have also shown that HD-AM can be at least as efficacious as cryopreserved AM when used as a substrate for ocular surface reconstruction [23].

The technique of HD-AM transplantation is increasingly being used for ocular surface reconstruction [8, 10]. It can be maintained at room temperature and cut easily to the desired size and shape just before application [7]. It is useful in the covering of wide ocular surface defects such as in the case of large or double-headed pterygium. Furthermore, many surgeons have stated that care for HD-AM is simple, and its use may lead to shorter operating times. When compared to available synthetic biomaterials and animal-derived alternatives, it has good mechanical properties that allow it to be directly surgically sutured [7].

In our study, the recurrence rate in the HD-AM group was significantly lower (5.06%) than recurrence rates in the CG group (20.97%; *P* = 0.003). The reported recurrence rates with amniotic membrane transplantation vary between 3.8 and 40.9% [24–26]. Inhibition of pathological neovascularization, prevention of excessive inflammation, and promotion of conjunctival epithelialization are the main reasons for the effectiveness of AM in pterygium surgery; therefore, use of HD-AM might help these processes and reduce recurrence of the condition [27–29].

It is interesting that the mean time to recurrence was 10.3 months in the HD-AM group as against 3.4 months in the CG group. This result correlated with a study by Kocamis, in which the average recurrence time was 4.5 months in conjunctival autografting [30]. This phenomenon suggests that conjunctival autografting provides a source of conjunctival epithelium and may eventually breach, whereas HD-AM seems to play a role in inhibiting the involvement of progenitor cells in pterygium recurrence [31].

To date, graft edema, necrosis of the graft, inclusion cysts, subconjunctival hematoma, Tenon’s granuloma, corneal narrowing, and Dellen ulcers have been reported to be the most common postoperative complications of primary pterygium [32, 33]. Three cases of Dellen ulcer and two cases of Tenon’s cyst were observed in the CG group in our study; whereas, none of these complications were observed in the HD-AM group during the follow-up period.

In addition to minimizing recurrence rates and surgical complications, it is expected that patients treated with HD-AM will have reduced postoperative pain and discomfort relative to conjunctival autograft surgery. These findings could be attributed to membrane covering of the corneal epithelial defect in addition to reducing inflammation [34].

## Conclusions

In summary, HD-AM transplantation may be a superior treatment in primary pterygium owing to lower recurrence rate, shorter surgical times, and no major complications other than conjunctival autografting. HD-AM is a human-derived material that has potential safety issues in the context of viral and prion infection. Further studies are needed to reevaluate the safety and efficacy of HD-AM for excision of primary pterygium.

## Abbreviations

AM: amniotic membrane
CG: conjunctival autografting
HD-AM: hyperdry amniotic membrane
TGF-β1: Transforming growth factor beta-1
UV: ultraviolet
